# Selenium Status and Cardiovascular Risk Profile in Healthy Adult Saudi Males

**DOI:** 10.3390/molecules14010141

**Published:** 2008-12-31

**Authors:** Eman M. Alissa, Waqar H. Ahmed, Nabeel Al-ama, Gordon A. A. Ferns

**Affiliations:** 1 Faculty of Medicine, King Abdul Aziz University, Jeddah, Kingdom of Saudi Arabia; 2 Postgraduate Medical School, Faculty of Health & Medical Science, University of Surrey, Guildford, Surrey GU2 7WG, UK; 3 Department of Cardiology, King Fahd Armed Forces Hospital, Jeddah, Kingdom of Saudi Arabia; 4 Department of Medicine, King Abdul Aziz University Hospital, Jeddah, Kingdom of Saudi Arabia

**Keywords:** Selenium, Iodine, Thyroid, Glutathione peroxidase, Subclinical hypothyroidism, Coronary risk factors, Lipoprotein (a), Saudi Arabia.

## Abstract

The purpose of this research was to investigate the relationship between selenium levels, thyroid function and other coronary risk factors in 140 Saudi subjects without overt coronary heart disease stratified by age. Demographic data and serum fasting lipid profile, glucose, thyroid function tests, selenium status and dietary intake was assessed. The relationships between selenium status, thyroid function and cardiovascular risk factors were assessed by univariate and multivariate analysis. The results showed that thyroid hormone levels did not differ with age. Erythrocyte glutathione peroxidase (GPx) levels were significantly higher in the youngest *vs*. oldest tertile (p<0.0001). Selenium and iodine intake did not differ significantly with age tertile, but the average intake for the population sample was below the estimated average requirements for both elements. Serum lipoprotein (a) concentrations correlated with selenium (r = 0.417, p<0.0001) and TSH (r = 0.172, p<0.05). After adjustment for confounding variables; serum fT_4_ and erythrocytes GPx remained significant determinants of serum TSH levels, whilst serum selenium and TSH were determinants of serum fT_4_ levels. Serum Lp(a), a coronary risk factor, was strongly related to measures of selenium status. A significant relationship between measures of selenium status and thyroid function was found. Serum Lp(a) a known risk factor for cardiovascular disease was also related to selenium status in our population.

## Introduction

Overt hypothyroidism is associated with dyslipidemia, hypertension, and an increased risk of cardiovascular disease (CVD) [1,2]. Lesser degrees of thyroid dysfunction may also be associated with increased coronary risk. Subclinical hypothyroidism, defined as an asymptomatic state characterized by normal serum concentrations of free thyroxine (fT4) and elevated serum concentrations of thyroid-stimulating hormone (TSH) [3], is found in high prevalence in elderly women [4]; its features include abnormal lipid metabolism [5,6,7], increased levels of plasma lipoprotein (a) (Lp (a)) [8,9,10], cardiac dysfunction [11], increased levels of oxidized LDL (ox-LDL) [12,13] and neurological and mental dysfunction [14]. Several cross-sectional studies have suggested that subclinical hypothyroidism confers an elevated risk of CVD [15,16,17], though this has been contested [18,19,20]. Possible atherogenic factors other than hyperlipidemia, such as elevated CRP, elevated tissue factor activities have been suggested [21,22]. Subclinical hypothyroidism itself has been identified as an independent risk factor for aortic atherosclerosis and myocardial infarction in elderly women [16] and variation of thyroid function within the normal range may influence the presence and severity of coronary atherosclerosis [23], even though the level of evidence is probably insufficient [24].

The importance of selenium for human health (reviewed in [25]) has been recognized for many years. One role of selenium is the formation of selenocysteine, located in the catalytic centre of all selenoenzymes, which are involved in the maintenance of redox balance in cellular and extracellular compartments. Selenium also plays a major role in T4 conversion to T3. The activity of all 3 known deiodinases, D1–D3, is dependent on selenium [26,27,28]. The importance of selenium in thyroid hormone metabolism is also reflected by the fact that the thyroid is the organ with the highest selenium content [29,30]; and the levels of selenium remain higher in the thyroid compared with other tissues even when deficiency occurs [31]. It has been shown that in critically ill patients, selenium supplementation leads to an earlier normalization of plasma T3 levels compared with controls [32] and that low plasma selenium levels correlate with low T3 levels [33]. From these studies, it has been concluded that the main cause of low T3 levels might be a reduced level of the selenoenzyme iodothyronine deiodinase (ID-I) activity.

Up to 80% of circulating T_3_ is produced by the activity of the selenium-containing enzyme ID-I. Thus, thyroid hormone metabolism may be affected by deficiencies of both selenium and iodine. In severe selenium deficiency hepatic ID-I activity decreases substantially, leading to an increase in plasma T_4_ concentrations. However plasma T_3_ concentrations are reported to be only slightly decreased in selenium deficiency due to several compensatory mechanisms including the maintenance or increase in thyroidal ID-I activity and decreased hepatic T_3_ degradation [34,35]. 

Furthermore, the selenoprotein glutathione peroxidase (GPx) may protect the thyroid gland from oxidative damage due to any excess hydrogen peroxide (H_2_O_2_) produced during thyroid hormone synthesis [36]. Thus, selenium deficiency may exacerbate some effects of iodine deficiency and may have a role in the aetiology of iodine-deficiency disorders [37,38]. Hence the combination of low selenium status and mild iodine deficiency may have a clinically significant impact. A number of human studies have shown alterations in the T_3_:T_4_ ratio is associated with low selenium and iodine status [39,40,41]. Therefore, it may be important to determine whether there are any detrimental effects within a population associated with the combination of marginal selenium and iodine status. 

The interaction between iodine and selenium is of particular interest in some countries like New Zealand, whose inhabitants have relatively low intakes of both trace elements. The selenium status is lower than that of residents of many other countries [42], even though blood selenium of New Zealanders has increased in recent years due to changes in dietary patterns and increases in selenium concentrations of some foods [43]. Despite iodization of salt since the 1930s, there has been a decrease in iodine status of New Zealanders during the past two decades. We have recently demonstrated that low serum selenium concentrations were highly associated with atherosclerosis in a Saudi male population after adjusting for other risk factors, including erythrocyte GPx [44]. In the current study, we have examined whether thyroid function is related to indices of selenium status in Saudi males without overt CHD, and whether selenium status influences the levels of several established cardiovascular risk factors including serum Lp (a). Our underlying hypothesis was that selenium status may influence thyroid function and this may in turn affect coronary risk. 

## Results and Discussion

### Demographic, anthropometric, biochemical and clinical characteristics

The demographic and anthropometric data of the subjects are shown in Table 1. The frequency of coronary risk factors; hypertension, diabetes mellitus and obesity increased with age tertiles (p<0.05). There was no clear distinction between age groups for smoking habit and physical activity. However a significantly higher proportion of the young and middle aged group had a positive smoking habit compared to the older group of subjects (p<0.0001). A large proportion (approximately 30%) of the combined group of middle and older individuals had a history of diabetes mellitus. Fasting glucose levels rose with age, consistent with the high frequency of diabetes in the oldest group (p<0.0001).

**Table 1:** Demographic and anthropometric characteristics of the subjects investigated divided into tertiles by age.

Variable	Age groups (years)			p
	≤ 30	31-48	≥ 49	
n=	46	47	47	
Age (years)	23.4 (16-30)	38.3 (31-48)	61.5 (49-87)	
Height (cm)	169.9 ± 0.9	167.9 ± 1.2	168.2 ± 1.2	NS
Weight (Kg)	75.8 ± 2.5	79.6 ± 2.3	81.3 ± 2.2	NS
Body mass index (Kg/m2)	26.3 ± 0.8	28.1 ± 0.7 §	28.8 ± 0.8 *	<0.05
Systolic blood pressure (mm Hg)	122.4 ± 1.5	127.9 ± 2.0	125.3 ± 3.2	NS
Diastolic blood pressure (mm Hg)	78.8 ± 1.1	83.1 ± 1.3 §¶	79.4 ± 1.6	<0.05
Hypertensive, n(%)	10 (22)	15 (32)	24 (51)	<0.05
Dyslipidemia, n(%)	37 (80)	44 (94)	44 (94)	NS
Diabetics, n(%)	0 (0)	14 (30)	17 (36)	<0.0001
Body mass index, n(%)				
Normal	23 (50)	11 (23)	14 (30)	
Overweight	13 (28)	22 (47)	12 (25)	<0.05
Obese	10 (22)	14 (30)	21 (45)	
Family History, n(%)				
Heart disease	11 (24)	12 (26)	10 (21)	NS
Diabetes mellitus	25 (54)	25 (53)	17 (36)	NS
Smoking status, n(%)				
Never	30 (65)	19 (40)	27 (57)	
Former	4 (9)	6 (13)	15 (32)	<0.001
Current (< 20 cigarette)	4 (9)	7 (15)	1 (2)	
Current (≥ 20 cigarette)	8 (17)	15 (32)	4 (9)	
Physical activity, n(%)				
< 3 times/week	13 (28)	14 (30)	12 (25)	NS
≥ 3 times/week	33 (72)	33 (70)	35 (75)	

Age is presented as mean (ranges). Numeric data are presented as mean ± SEM for normally distributed data and categorical data as number and percentage. Categorical data were compared by χ^2^ test, continuous variables were compared by Kruskal-Wallis test. Diabetes was defined as a known history of diabetes mellitus (fasting blood glucose >7 mmol/L) or treatment with insulin or oral hypoglycemic agents. Dyslipidaemia was defined as total cholesterol level ≥ 5.2 mmol/L, a LDL-C ≥ 3.36 mmol/L, and/or a HDL-C< 1.04 mmol/L. Total cholesterol/HDL-C ratio was used as an index of CVD risk. Hypertension was defined as a systolic blood pressure above 140 mmHg, or diastolic blood pressure above 90 mmHg, respectively, or current use of antihypertensive medication. NS: not signifiant, * P<0.05 (≤ 30y versus ≥ 49y groups),, ¥ P<0.001 (≤ 30y versus ≥ 49y groups), § P<0.05 (≤ 30y versus 31-48y groups), # P<0.001 (≤ 30y versus 31-48y groups), P<0.05 (31-48y versus ≥ 49y groups ), † P<0.001 (31-48y versus ≥ 49y groups).

### Lipid profile data

**Figure 1:**
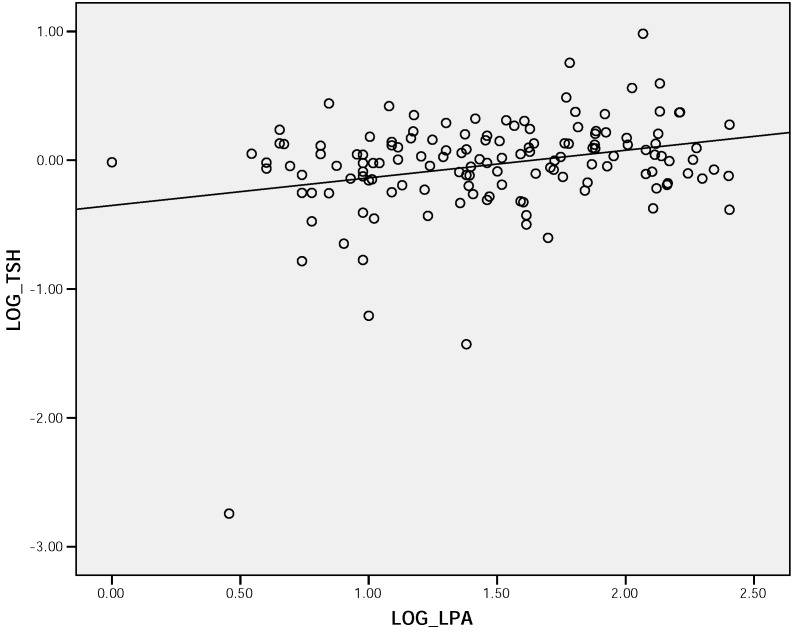
Scatter plot demonstrating correlation between serum Lp (a) concentration and serum TSH among the whole population (r = 0.172, p <0.05).

The biochemical data for the groups segmented by age are shown in Table 2. A large proportion of all age groups had a fasting serum total and LDL-cholesterol above NCEP ATP III guidelines. The middle age group appeared to have the worst overall lipid profile, with higher mean LDL cholesterol (p=0.055) and triglycerides (p<0.05). The distribution of serum Lp (a) concentrations were statistically different between the three groups (p<0.0001), with higher values in older groups and lower values in the young group. Moreover, in the population as a whole, a significant correlation was observed between Lp (a) and TSH (Figure 1). 

### Thyroid hormones

Thyroid hormone status did not differ significantly between age tertiles. However, there was a gradual increase in the mean value of serum TSH with age group (Table 2). Although mean serum fT_4_ was lowest for the youngest age group of subjects, the differences did not reach statistical significance (p>0.05). 

**Table 2:** Biochemical characteristics of the subjects investigated divided into tertiles by age.

Variable		Age groups (years)			p
		≤ 30	31-48	≥ 49	
n=		46	47	47	
Total cholesterol [mmol/L]		5.38 ± 0.19	6.01 ± 0.20	5.69 ± 0.23	NS
Total cholesterol					
≥ 5.2 mmol/L, n(%)		25 (54)	31 (66)	27 (57)	NS
Triglycerides [mmol/L]		1.14 (0.86-1.5)	1.55 (0.95-2.5) §	1.34 (0.77-2.1)	<0.05
Triglycerides ≥					
1.7 mmol/L, n(%)		9 (20)	21 (45)	16 (34)	<0.05
HDL-C [mmol/L]		1.45 ± 0.09	1.49 ± 0.09	1.29 ± 0.07	NS
HDL-C					
< 1.04 mmol/L, n(%)		14 (30)	12 (26)	12 (26)	NS
LDL-C [mmol/L]		3.67 ± 0.21	4.2 ± 0.19	4.1 ± 0.22	NS
LDL-C					
≥ 3.36 mmol/L, n(%)		26 (57)	33 (70)	31 (66)	NS
Atherogenic index (TC/HDL)		4.40 ± 0.32	4.6 ± 0.29	4.9 ± 0.29	NS
Lipoprotein (a) [mg/dL]		14.8 (9.38-35.48)	40 (12.3-74.5) §	69.5 (24-134) ¥	<0.0001
Glucose [mmol/L]					
	(Whole group)	5.37 (4.9-5.8)	5.80 (5.2-7.2) #	6.10 (5.4-9.3) ¥	<0.0001
	(Diabetics)	-	9.2 (7.9-12.1)	10.2 (8.6-12.0)	NS
	(Non-diabetics)	5.37 (4.9-5.8)	5.46 (5.2-5.9)	5.5 (5.2-6.0)	NS
TSH (mIU/L)		1.03 ± 0.08	1.2 ± 0.14	1.4 ± 0.21	NS
fT_4_ (pmol/L)		23.9 ± 0.44	23.9 ± 0.56	25.1 ± 0.59	NS
Subclinical hypothyroidism TSH>					
4.2 mIU/L & normal fT4, n(%)		0 (0%)	1 (2%)	1 (2%)	NS
Serum Selenium (μmol/L)		0.40 ± 0.04	0.48 ± 0.06	0.51 ± 0.04	NS
Urine Selenium (μmol/mol creatinine)		1.48 ± 0.21	1.29 ± 0.19	1.07 ± 0.12	NS
Erythrocytes GPx (IU/ gm Hb)		113.4 ± 8.6 ¥	111.5 ± 13.6	72.5 ± 7.4	<0.05

Numeric data are presented as mean ± SEM for normally distributed data and as median (interquartile range) for non-normally distributed data. Continuous variables were compared by Kruskal-Wallis test. fT_4_: free thyroxine, GPx: glutathione peroxidase, HDL-C: high density lipoprotein cholesterol, LDL-C: low density lipoprotein cholesterol, NS: not significant, TSH: Thyroid Stimulating Hormone. ¥ P<0.001 (≤ 30 y versus ≥ 49 y groups), § P<0.05 (≤ 30 y versus 31-48 y groups), # P<0.001 (≤ 30 y versus 31-48 y groups).

### Selenium status

**Figure 2:**
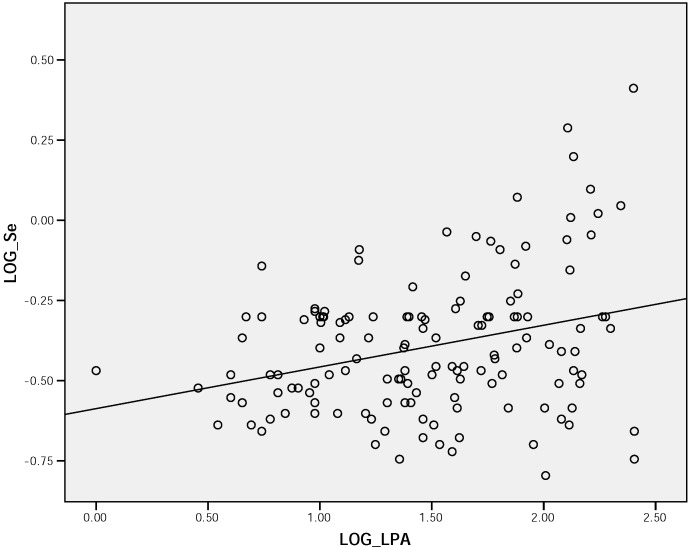
Scatter plot demonstrating correlation between serum Lp (a) concentration and serum selenium among the whole population (r = 0.417, p <0.0001).

**Figure 3:**
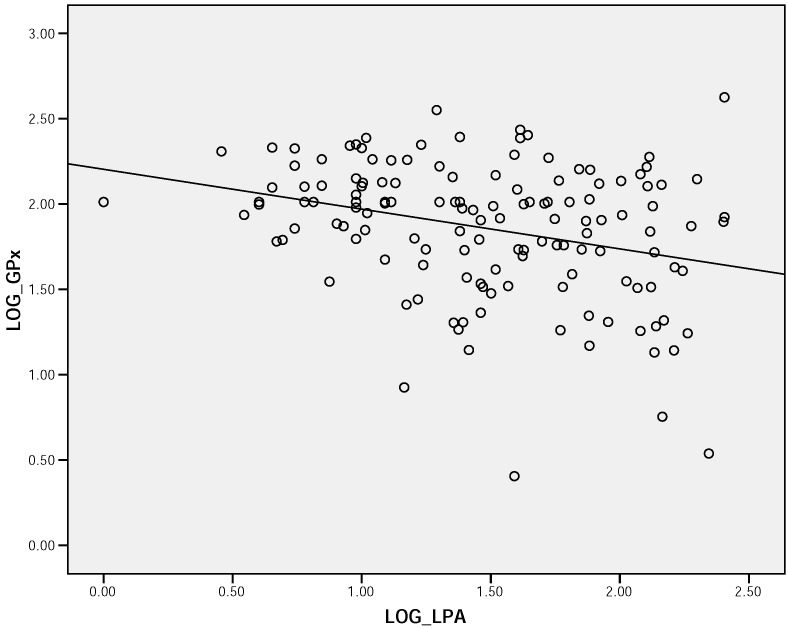
Scatter plot demonstrating correlation between serum Lp (a) concentration and erythrocytes GPx among the whole population (r = - 0.304, p <0.0001).

Whilst urine selenium concentrations fell, and serum selenium concentrations rose with age tertiles, as indicated in Table 2, these just failed to reach statistical significance (p>0.05). However erythrocyte GPx levels were significantly higher in the younger age tertile, compared to the oldest (p<0.0001). The correlation coefficients between each marker of selenium status and thyroid hormones, and lipid profile parameters are shown in Table 3. Interestingly, Figure 2 illustrates a strong correlation between serum Lp (a) concentration and serum selenium among the whole population. An inverse correction was also observed between Lp(a) and erythrocytes GPx concentrations (Figure 3).

**Table 3:** Correlation coefficients between selenium status and thyroid hormones variables and lipid profile in the subjects investigated (n=140).

Selenium status parameters		r	p
Serum Selenium	Erythrocytes GPx	0.178	0.036
	Serum fT_4_	0.274	0.001
	Urine Selenium	0.537	<0.0001
	Serum Lp (a)	0.417	<0.0001
Urine Selenium	Total Cholesterol	0.218	0.01
	LDL-Cholesterol	0.239	0.004
	Fat intake	0.206	0.015
Erythrocytes GPx	Serum TSH	- 0.210	0.013
	Serum fT_4_	0.211	0.012
	Total Cholesterol	0.201	0.017
	HDL-Cholesterol	0.180	0.033
	Serum Lp (a)	- 0.304	<0.0001
	Energy intake	0.183	0.031
	Fat intake	0.269	0.001
	SFA intake	0.327	<0.0001

fT_4_: free thyroxine, GPx: glutathione peroxidase, Lp (a): Lipoprotein (a), MUFA: monosaturated fatty acids, PUFA: polysaturated fatty acids, SFA: saturated fatty acids, TSH: Thyroid Stimulating Hormone

**Table 4:** Dietary characteristics of the subjects investigated divided into tertiles by age.

Nutrient	RNI	Age groups (years)			p
		≤ 30	31-48	≥ 49	
n=		46	47	47	
On a diet, n(%)		4 (9)	11 (23)	10 (21)	NS
Energy (Kcal)	2755 (15-18)				
	2550 (19-59)	2188.7 ± 66.2 ¥	2010.6 ± 64.7	1840.0 ± 62.8	<0.001
	2380 (60-74)				
	2100 (75+)				
Total fat (gm)%		92.6 ± 3.01 *	85.0 ± 3.69	79.5 ± 3.59	<0.05
					
of energy	30 %	38.3 ± 0.72	38.1 ± 1.02	38.5 ± 0.88	NS
Cholesterol (mg)		313.2 ± 15.5 ¥	272.1 ± 18.6	238.1 ± 14.9	<0.05
Categories of	< 200 mg	2 (4)	15 (32)	21 (45)	<0.001
cholesterol intake n(%)	≥ 200 mg	44 (96)	32 (68)	26 (55)	
SFA (gm)%		32.7 ± 1.3 §¥	27.8 ± 1.64	26.0 ± 1.42	<0.05
of energy	10%	15.0 ± 0.40	13.7 ± 0.55	13.9 ± 0.51	NS
MUFA (gm)%		31.8 ± 1.0	30.1 ± 1.37	28.0 ± 1.29	NS
of energy	10%	14.7 ± 0.34	15.1 ± 0.48	15.1 ± 0.38	NS
PUFA (gm)%		18.4 ± 0.85	18.2 ± 0.83	17.2 ± 0.96	NS
of energy	10%	8.4 ± 0.32	9.1 ± 0.32	9.3 ± 0.40	NS
P:S ratio	NA	0.58 ± 0.03	0.71 ± 0.04 §	0.72 ± 0.05 *	<0.05
Selenium (μg)	75 μg	47.9 ± 2.2	46.1 ± 2.7	41.7 ± 1.8	NS
Se intake <EAR	< 57.7 μg	40 (87)	41 (87)	40 (85)	NS
Iodine (μg)	150 μg	133.8 ± 8.7	133.9 ± 8.7	115.8 ± 5.9	NS
I_2_ intake <EAR	<115.4 μg	15 (33)	25 (53)	25 (53)	NS

Numeric data are presented as mean ± SEM and categorical data as number and percentage. Categorical data were compared by χ^2^ test, continuous variables were compared by Kruskal-Wallis test. EAR: estimated average requirements, MUFA: monounsaturated fatty acids, NS: not significant, P:S ratio: polyunsaturated fatty acids: saturated fatty acids ratio, PUFA: polyunsaturated fatty acids, RNI: reference nutrient intake, SFA: saturated fatty acids, * P<0.05 (≤ 30y versus ≥ 49y groups), ¥ P<0.001 (≤ 30y versus ≥ 49y groups), § P<0.05 (≤ 30y versus 31-48y groups)

### Dietary intake

In our population sample, there was a high intake of total- and saturated- fat, and cholesterol. Dietary total fat, saturated fat and cholesterol were all highest in the youngest age group (p<0.05) (Table 4). This could be related to the adoption of Western-style dietary habits. Total calorie intake decreased with age group; the proportion of subjects consuming excessive or adequate dietary calories was highest in the youngest group of subjects. This may be explained in part by the lower proportion of these individuals on a diet (9%). The mean intake of total fat and cholesterol was above recommended values for all age groups. Subjects in the youngest tertile for age had the lowest PUFA: SFA ratio of all age groups (p<0.05). In general, the intake of selenium and iodine showed no statistical difference between the age tertiles. However, the intake of a large proportion of the population sample (86% with selenium intake and 46% with iodine intake) was well below the estimated average requirement (EAR) values for both elements (Table 4).

### Multivariate analysis

Adjustment for possible confounding variables, including dietary intake of selenium and iodine levels to identify independent determinants of serum thyroid hormone levels, was undertaken for the data of the 140 individuals for all variables that showed a univariate relationship (p <0.01) and entered into multiple regression models (Table 5). In the first model with serum TSH as the dependent variables, the variables namely age, diastolic blood pressure, serum TC, serum TG, serum fT4, serum Lp(a), erythrocytes GPx and dietary cholesterol were included and in the second model with serum fT4 as the dependent variables, the variables: diastolic blood pressure, serum Se, serum TSH, serum Lp(a), and dietary selenium were included. 

**Table 5:** Multiple regression analysis between thyroid function test and the predicting risk factors in the subjects investigated (n=140).

Thyroid hormones (dependent variable)	Risk factors (Predictors)	β	95% CI limit for β		p
Serum TSH	Serum fT_4_	0.056	0.009	0.104	0.02
	Erythrocytes GPx	-0.002	-0.005	0.0	0.047
		**Total R^2^ = (0.285)^2^ = 8.1%**			
Serum fT_4_	Serum selenium	3.087	1.287	4.887	0.001
	Serum TSH	0.804	0.250	1.358	0.005
		**Total R^2^ = (0.357)^2^ = 12.8%**			

95% CI: 95% confidence interval, GPx: glutathione peroxidase, TSH: thyroid stimulating hormone

The best fitting stepwise multiple regression model with serum TSH level as a dependent variable showed that serum fT_4_ and erythrocytes GPx remained significant determinants and together they explained 8.1% of the variation (R^2^) in serum TSH level. Although erythrocytes GPx levels were found to be inversely correlated to serum TSH (r = - 0.210, p <0.05), multivariate analysis showed that the relationship was statistically independent of other factors entered into the model and alone predicting 2.7% of the variation in serum TSH level.

Using a similar approach for serum fT_4_ as a dependent variable in another stepwise multiple regression model that explained 12.8% of the variation (R^2^) in its concentration, serum selenium and TSH levels were found to be independent predictors in which serum selenium alone explained 5.3% of the variation. To the best of our knowledge, this is the first study reporting the association between measures of selenium status, including dietary intake, and indices of thyroid function and coronary risk. 

### Demographic and Biochemical data

There have been previous reports of a high prevalence of coronary risk factors in Saudi Arabia [45], and our findings in this study accord with this. In our sample of Saudi males, there was a high prevalence of classical coronary risk factors, including dyslipidaemia, diabetes mellitus, hypertension, obesity and a positive smoking habit. There were significant differences in the distribution of these risk factors between the age groups.

### Selenium and Thyroid hormone status

Previous case-control and cross-sectional studies on the association between subclinical hypothyroidism, selenium status and CHD are inconsistent [15,20,46,47,48,49,50]. The overall prevalence of subclinical hypothyroidism in our sample was 4%, similar to the previously reported prevalence in the general population [51].

The higher risk of CVD associated with hypothyroidism may be because of its association with elevated serum LDL-C concentrations [52,53]. It has also previously been reported that concentrations of serum Lp (a), another established CVD risk factor, are raised in subclinical hypothyroidism [10,54], and in this present study, serum selenium (r=0.417, p<0.0001) and erythrocyte GPx (r=- 0.304, p<0.0001) were both strongly associated with serum Lp (a) concentrations. Univariate analysis has shown that serum Lp (a) level was significantly correlated with both serum TSH and fT_4_ levels. However, multivariate analysis showed that the relationship was not totally independent of other factors entered into the model and therefore lost its statistical significance.

It has been postulated that diabetes may affect selenium status in hypothyroidism patients, compared to their pretreatment levels with hormone replacement therapy [18]. Our data were reanalyzed after excluding the diabetic subjects in order to check whether diabetes was indeed associated with selenium status in this population, but this factor was not found to be of significance (p>0.05) and therefore, it is unlikely that diabetes affected the validity of our results.

Overall, our population sample had low indices of selenium and iodine status. However, the indices we have measured may not reflect the total body selenium content, nor reflect the tissue specific distribution of the selenoenzymes [55]. For example the thyroid contains more selenium per gram of tissue than any other organ [56] and this may be less affected during dietary selenium insufficiency.

### Dietary intake data

Thyroid hormone metabolism is sensitive to the total dietary energy and carbohydrate content [57] and it may also be affected by other dietary components including iodine [58] and iron [59]. There is a possibility of synergistic effects of selenium and iodine deficiencies on thyroid function in our population sample given the high prevalence of individuals consuming both selenium and iodine below the EAR (86% and 46% respectively). High dietary iron content might also interfere with selenium bioavailability [60]. The strong associations between indices of selenium status and indices of thyroid function are consistent with the hypothesis that GPx may protect the thyroid follicular cells from oxidative damage following H_2_O_2_ release during thyroid hormone synthesis [38]. This might be illustrated by the positive association between erythrocyte GPx and the components of atherogenic diet: energy, total fat and saturated fat (Table 3). The effect of dietary fatty acids on concentrations of serum cholesterol and particularly LDL-C is well documented [61]. Previous studies have demonstrated positive correlation between high levels of lipid peroxidation and atherogenic lipid profile [62].

### Multiple regression models

Multiple regression analysis was performed to adjust the data for the possible effects of confounding factors, between markers of thyroid status and coronary risk factors in the whole population. Surprisingly, dietary selenium and iodine intake were no longer related to thyroid status in the population after this adjustment. 

According to these models, serum TSH can be predicted by erythrocyte GPx concentration and serum fT_4_ can also be predicted by serum selenium level. This is consistent with previous reports on the protective effects of GPx against the oxidative damage of H_2_O_2_ [38]. Although GPx require selenium for its function and play an important role in thyroid hormone metabolism, available data suggest that the effect of selenium deficiency on thyroid function is relatively modest and the functional effects of low selenium status on thyroid function in humans have so far not been fully characterized [37].

## Conclusions

Thyroid disorders are known to influence lipid metabolism and are common in dyslipidemic patients. In our population sample of Saudi males without clinically overt CVD we found a significant relationship between measures of selenium status and tests of thyroid function. Although the mild perturbation of selenium status seen in our population sample may not be expected to affect thyroid function in isolation, the combination of low selenium status and mild iodine deficiency may indeed have a significant effect. The associations between selenium status and thyroid function may explain, in part, the inconsistent reports of an association between thyroid and selenium status and CVD. The impact of selenium deficiency on thyroid function is likely to be dependent on other factors such as iodine sufficiency that may vary with the population under investigation. Additional research should be done to determine whether this association can be confirmed in a prospective study.

## Experimental

### Subject recruitment

One hundred and forty male subjects without clinically evident coronary disease were recruited from the Outpatients’ departments of King Abdul Aziz University Hospital and The King Fahad Armed Forces Hospital, Jeddah, Kingdom of Saudi Arabia based on their cardiologists’s decision. They were usually referred for risk factor modification, principally hyperlipidemia, hypertension, and diabetes mellitus, but were free from overt coronary disease. The absence of CVD was confirmed by medical measurements performed routinely and they include ECG recording and blood cardiac enzymes. Their age varied between 16 and 87 years. On direct questioning, the subjects reported no history of CHD symptoms, and had no personal history of acute coronary syndrome (including prior acute coronary events, percutaneous transluminal coronary angioplasty (PTCA) or bypass surgery), a history of heart failure, vascular disease (i.e. peripheral vascular disease, cerebrovascular disease) documented in their medical notes. We also excluded those patients who had co-morbidities (malignancy, hepatic, or renal failure). No patient received any medication such as amiodarone, thyroid hormones or iodine-containing agents that could alter thyroid function, or those on treatment with statins, antioxidants or aspirin. The local ethics committee of the hospital approved the study.

### Blood samples

Fasting venous blood samples were taken after an overnight fast and placed into plain, or heparinized tubes. Tubes were centrifuged at 3000 x *g* for 10 min. The serum obtained was separated and frozen at -80^o^C until the time of analysis. Urine samples were collected in polyethylene containers and centrifuged at 3000 *g* for 10 minutes. The supernatant obtained was separated and frozen at -80^o^C until the time of analysis. 

### Demographic, anthropometric, biochemical and clinical characteristics

Subjects were interviewed to complete a questionnaire, concerning their demographic characteristics. These included: age, personal and family health history (premature heart disease, diabetes and dyslipidaemia), lifestyle habits (e.g. smoking habit and physical activity levels) and diet. Weight, body mass index (BMI), systolic and diastolic blood pressure were assessed for each subject using routine procedures. 

Dyslipidaemia was defined as total cholesterol level ≥ 5.2 mmol/L, a LDL-C ≥ 3.36 mmol/L, and/or a HDL-C< 1.04 mmol/L. Total cholesterol/HDL-C ratio was used as an index of CVD risk [63].

Hypertension was defined as a systolic blood pressure above 140 mmHg, or diastolic blood pressure above 90 mmHg, respectively, or current use of antihypertensive medication. 

Diabetes was defined as a known history of diabetes mellitus (fasting blood glucose >7 mmol/L) or treatment with insulin or oral hypoglycemic agents [64]. 

In subjects with negative history of consumption of thyroid or antithyroid drugs, lithium, estrogens or androgens during the previous month, “overt hypothyroidism” was defined as elevated TSH and low fT_4_, “subclinical hypothyroidism” as elevated TSH and normal fT_4_. The level of overt hypothyroidism was defined as both TSH>4.2 mIU/L and fT_4_<10.3 pmol/L, while that of subclinical hypothyroidism as both TSH>4.2 mIU/L and normal fT_4 _ [65]. However, no case of overt hypothyroidism was observed in our study.

BMI was calculated as weight in kg/height^2^ in m^2^, and classified into normal; defined as BMI<25 kg/m^2^, overweight as BMI = 25–29.9 kg/m^2^, obese as BMI≥30 kg/m^2^. 

Smoking habit was categorized as non-smoker, former smoker, and current smoker. Current smokers were further categorized into those who smoke <20 cigarettes/ day and those who smoke ≥20 cigarettes/day. 

Physical activity was self-graded by the participant according to the number of episodes of exercise undertaken per week and were categorized as active (≥3 times/week) or inactive (<3 times/week) according to the recommendations of the American Heart Association consensus statement on primary prevention of coronary diseases and from the USA Surgeon General’s report.

### Routine analytical methods

Glucose was measured enzymatically by a routine glucose oxidase method. Total cholesterol and triglycerides were measured enzymatically by a colorimetric end-point method. HDL cholesterol was measured using a phosphotungstate magnesium precipitation method. LDL was calculated using the Friedewald formula in samples where the triglycerides were <4 mmol/L [66].

Thyroid function test included only TSH and fT_4_ and were assayed using a competitive immunoassay commercial kit (BioKit, S.A., Spain). The following reference intervals were determined: TSH, 0.27–4.2 mIU/L; fT_4_, 0.84–1.42 ng/dl (12– 22 pmol/L). Intra- and inter-assay coefficients of variation were ≤5.6 and ≤9.1% for TSH, ≤3.8 and ≤5.6% for fT_4_, respectively. 

### Serum Lipoprotein (a) Assay

Serum Lp (a) concentrations were measured in duplicate, using an ELISA assay kit (Biopool, CA, USA) following the manufacturers recommended technique. Intra- and inter-assay coefficients of variation were <10% and <20% respectively.

### Erythrocyte glutathione peroxidase activity

GPx activity was measured in erythrocyte lysates as a functional assessment of selenium status, using Randox kits (Randox Laboratories Ltd, UK). The red blood cell suspensions were prepared from phosphate buffered saline (PBS) washed red blood cells after removal of plasma. The red cells were washed thrice with PBS at 4^o^C. Cells were then re-suspended to their original volume with PBS. Erythrocyte hemolysates were obtained by adding 4 volumes of de-ionized distilled water to 1 volume of red blood cell suspension. The lysate was frozen at -80^o^C until the time of analysis. 

Measurement of GPx activity was based on the method of Paglia and Valentine [67]. The activity was expressed in U/g of haemoglobin in erythrocytes. The intra-assay and inter-assay coefficients of variation for each variable was found to be < 8% and < 15% respectively.

### Trace element analysis

All reagents were of at least analytical grade and supplied by Sigma-Aldrich chemicals (Sigma-Aldrich Ltd, ON, Canada) unless indicated otherwise. All glass or plastic ware used for trace elements determination were cleaned by soaking overnight in 10% (v/v) hydrochloric acid, followed by thorough rinsing with de-ionized distilled water and drying. Aqueous solutions were made up in de-ionized distilled water.

Trace element content was measured by atomic absorption spectrophotometer on a SOLAAR M5 (Thermo Electron, Cambridge, UK) with a deuterium background corrector. Serum selenium was measured using a GF95 graphite furnace with auto-sampler. Argon gas was used as the purging gas. Urine selenium was measured using a VP90 continuous flow vapour system. Urine samples were used to prepare a standard curve in the standard addition method. To avoid matrix interference using the graphite furnace, the standard addition method for calibration was used. Serum samples were diluted (1:4) with 0.05% Triton-X 100 in 0.125% (v/v) nitric acid. Nickel in nitric acid was used as a matrix modifier in order to determine serum selenium content. For the hydride generation method, samples were digested with nitric, sulphuric, and perchloric acids to a final temperature of 310^o^C [68]. Hydride generation was carried out using a solution of 5% (w/v) of NaBH_4_ dissolved in 1% (w/v) NaOH solution and 1.5% (v/v) HCl. The urinary output of trace elements was expressed in mg/mol of creatinine. The intra-assay and inter-assay coefficients of variation for each variable was found to be < 8% and < 15% respectively.

### Assessment of dietary intake

Dietary intake over the previous year was assessed using a previously validated semi-quantitative food frequency questionnaire (FFQ) [70]. The nutrient database used was based on UK food composition tables together with food composition tables for use in East Asia and the United States handbook of food composition. The estimated dietary intake of all nutrients was calculated in terms of percentage recommended nutritional intake (%RNI for UK adults) for each individual, as there are no published data for a Saudi population. 

### Statistical analysis

Data are presented as mean and standard deviation for normally distributed data, or as median and interquartile ranges for non-parametric data. Variables that showed a skewed distribution were log transformed before analyses and then back transformed to their natural units for presentation. Statistical analyses were performed using ANOVA test for normally distributed parameters or Kruskall-Wallis test for non-normally distributed parameters to compare mean values of repeated measures of non-normally distributed parameters. If a significant difference was found, a Bonferroni corrections were made for multiple comparisons to determine differences between each pair of groups. A χ^2^ test was used for comparison of categoric data. 

Associations were also assessed using Pearson’s and Spearman’s correlation coefficients. Stepwise multiple regression analysis was used to model the association between each selenium status marker with all independent variables with p value up to 0.1 to demonstrate their contribution to subclinical hypothyroidism. A p <0.05 was considered statistically significant. All statistical analysis was carried out with SPSS, version 11.5 software.
